# Supplementary material for: A novel domain within the CIL regulates egress of IFITM3 from the Golgi and reveals a regulatory role of IFITM3 on the secretory pathway
Source: Life Sci Alliance. 2022 Apr 8;5(7):e202101174. doi: 10.26508/lsa.202101174 (PMC8994042; doi:10.26508/lsa.202101174)
Supplement: Supplementary file 1 [file LSA-2021-01174_SdataF1_F6.pptx]

## Slide 1
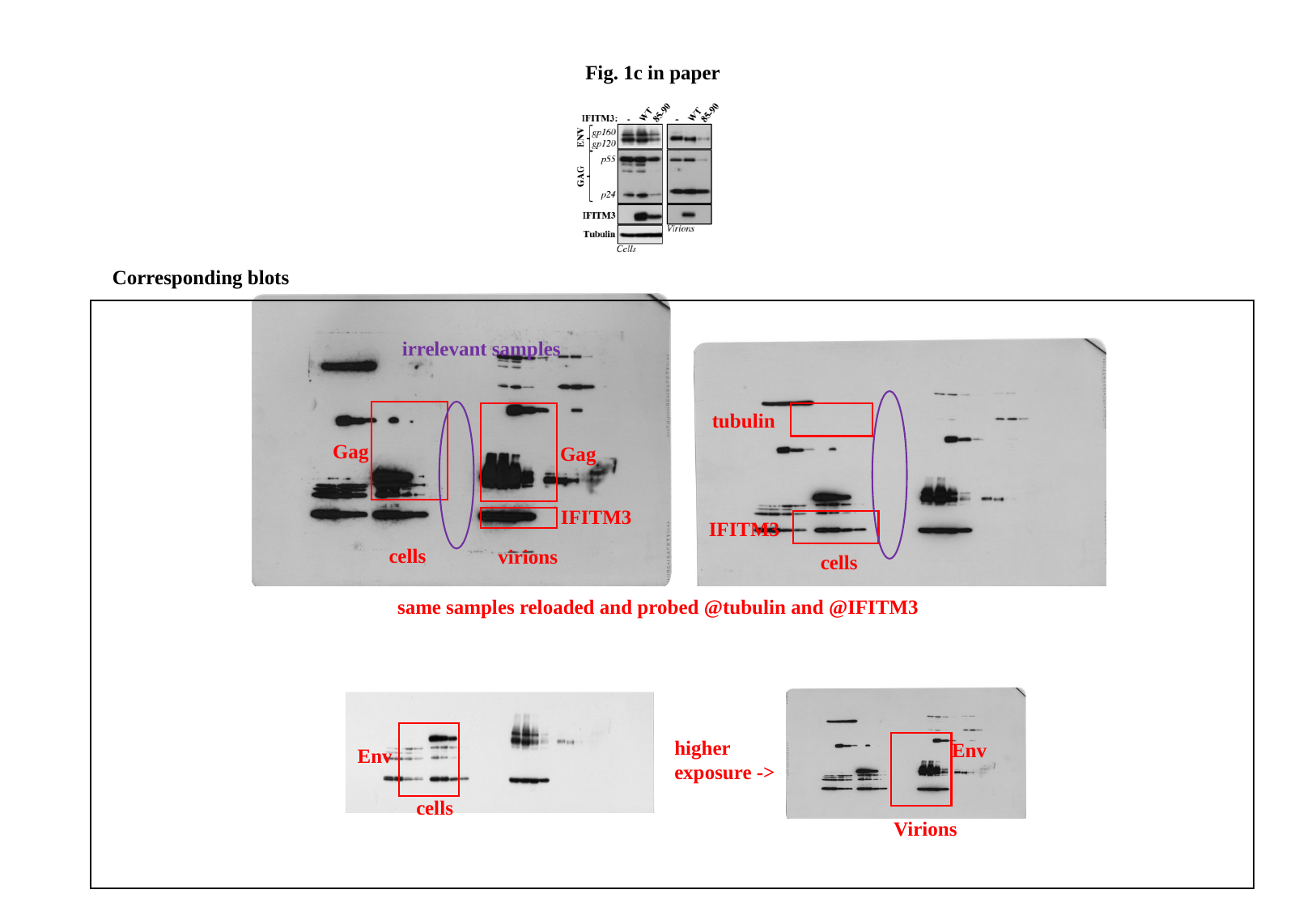

Fig. 1c in paper
Corresponding blots
irrelevant samples
tubulin
Gag
Gag
IFITM3
IFITM3
cells
virions
cells
same samples reloaded and probed @tubulin and @IFITM3
higher exposure ->
Env
Env
cells
Virions

## Slide 2
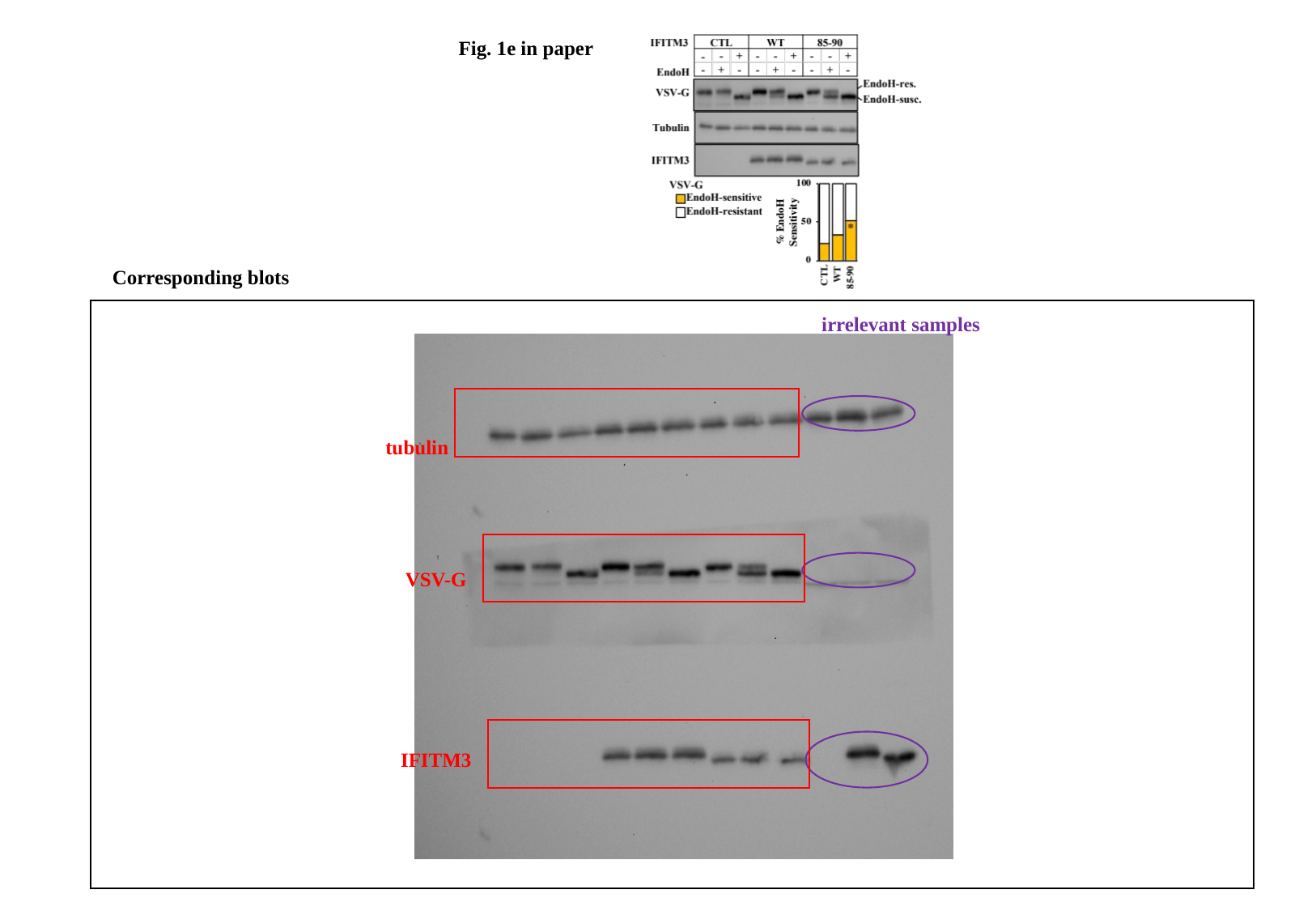

Fig. 1e in paper
Corresponding blots
irrelevant samples
tubulin
VSV-G
IFITM3

## Slide 3
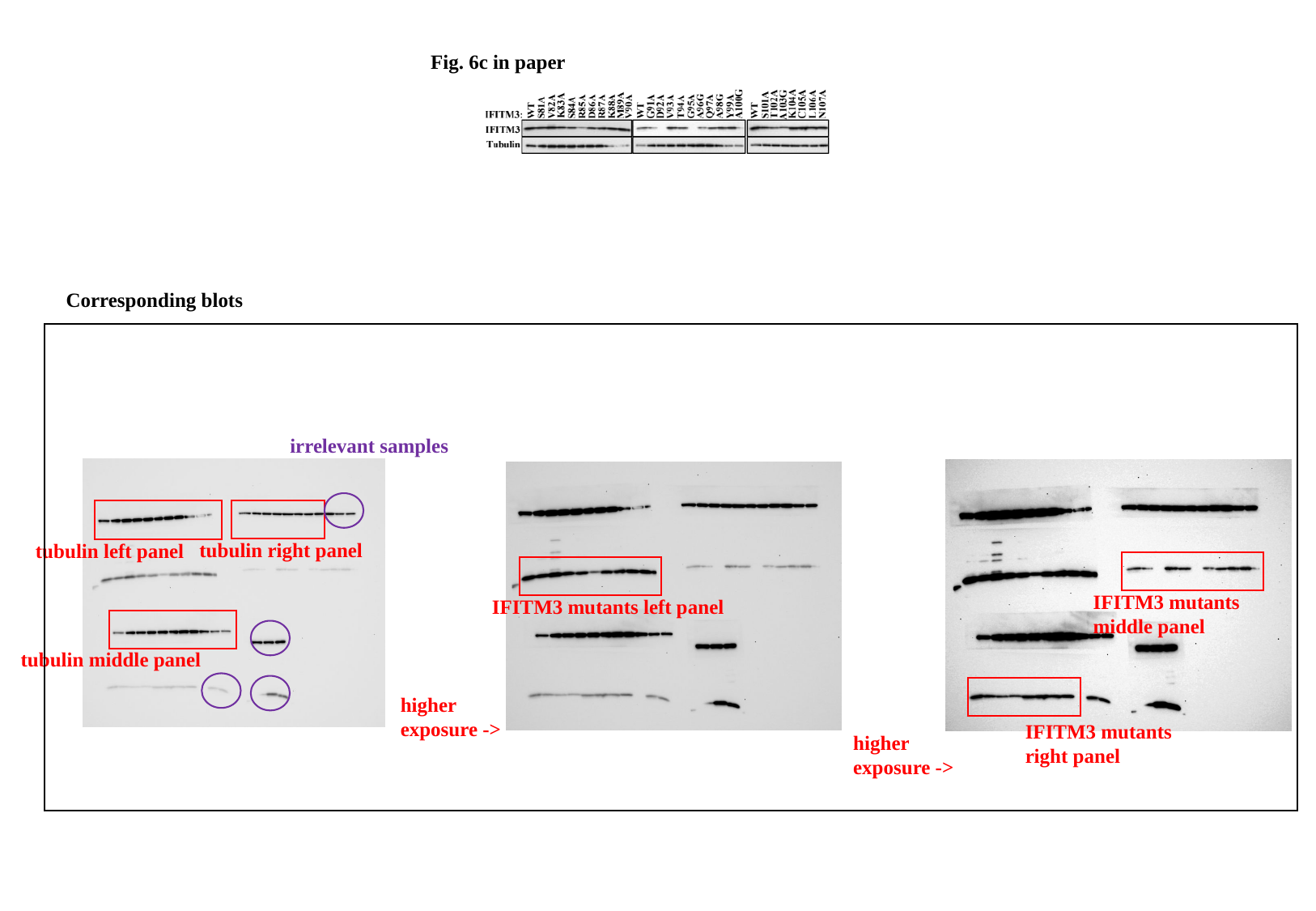

Fig. 6c in paper
Corresponding blots
irrelevant samples
tubulin right panel
tubulin left panel
IFITM3 mutants middle panel
IFITM3 mutants left panel
tubulin middle panel
higher exposure ->
IFITM3 mutants right panel
higher exposure ->
